# Solitary Neurofibroma of the Hypopharynx: A Case Report

**DOI:** 10.1002/ccr3.72739

**Published:** 2026-05-22

**Authors:** Matin Ghazizadeh, Vahid Ghasem Amooeian

**Affiliations:** ^1^ Department of Otolaryngology–Head & Neck Surgery Shahid Beheshti University of Medical Sciences, Taleghani Hospital Tehran Iran

**Keywords:** hypopharynx, neurofibroma, postcricoid, surgery, transoral

## Abstract

Although hypopharyngeal neurofibroma is extremely rare, it must be included in the differential diagnosis of submucosal hypopharyngeal masses. Magnetic resonance imaging defines the lesion's extent, and transoral excision provides safe and complete resection with rapid symptom resolution.

## 1 Introduction

Solitary neurofibroma is a benign peripheral nerve sheath tumor comprising Schwann cells, fibroblasts, and perineurial‐like cells. Though neurofibromas are common in the context of neurofibromatosis type 1 (NF1), true solitary neurofibromas unassociated with genetic syndromes are rare, especially within the upper aerodigestive tract [1]. Tumors arising in the hypopharynx are exceedingly infrequent, with only isolated case reports in the literature. Hypopharyngeal tumors often manifest with nonspecific symptoms, presenting diagnostic challenges, while their removal carries the risk of injury to adjacent critical structures such as the larynx, esophagus, and recurrent laryngeal nerve. Here, we describe the presentation, diagnostic work‐up, surgical management, and histopathological findings of a solitary neurofibroma in the posterior hypopharynx of a 67‐year‐old man, highlighting contemporary diagnostic and treatment considerations [2].

## 2 Case Report

A 67‐year‐old male with no prior significant medical history was referred with a 1‐year history of progressive hoarseness and intermittent dysphagia to solids that had steadily worsened over 3 months. He denied dyspnea, stridor, hemoptysis, odynophagia, weight loss, or a history of head and neck neoplasia. On physical examination, there was a barely perceptible dysphonia without inspiratory stridor. Neck palpation revealed no masses, lymphadenopathy, or cutaneous stigmata of NF1 (such as café‐au‐lait macules, axillary freckling, or subcutaneous nodules).

**FIGURE 1:**
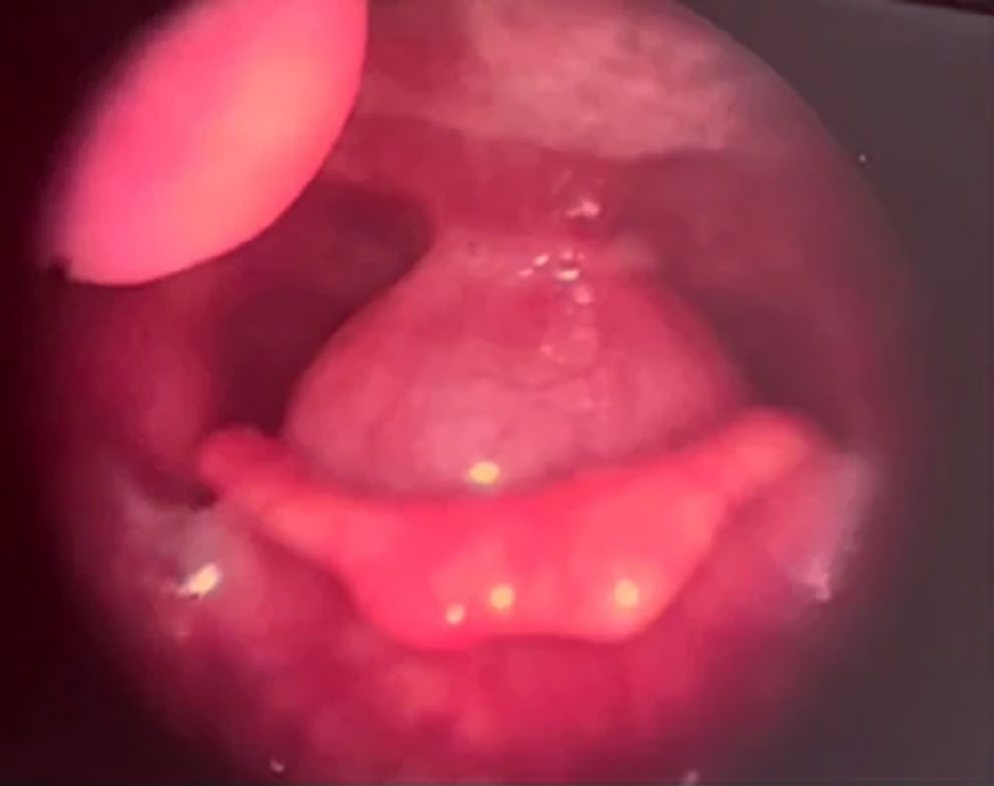
Rigid laryngoscopic view showing a smooth, pedunculated mass on the posterior pharyngeal wall at the level of the hypopharynx.

Rigid laryngoscopy demonstrated a smooth, well‐demarcated submucosal mass located on the posterior pharyngeal wall, just under the tip of the epiglottis (Figure 1). The mass did not encroach upon the true vocal cords; both vocal folds were mobile. There was no mucosal ulceration or surface abnormality, and the piriform sinuses remained patent.

### 2.1 Imaging Work‐Up

**FIGURE 2:**
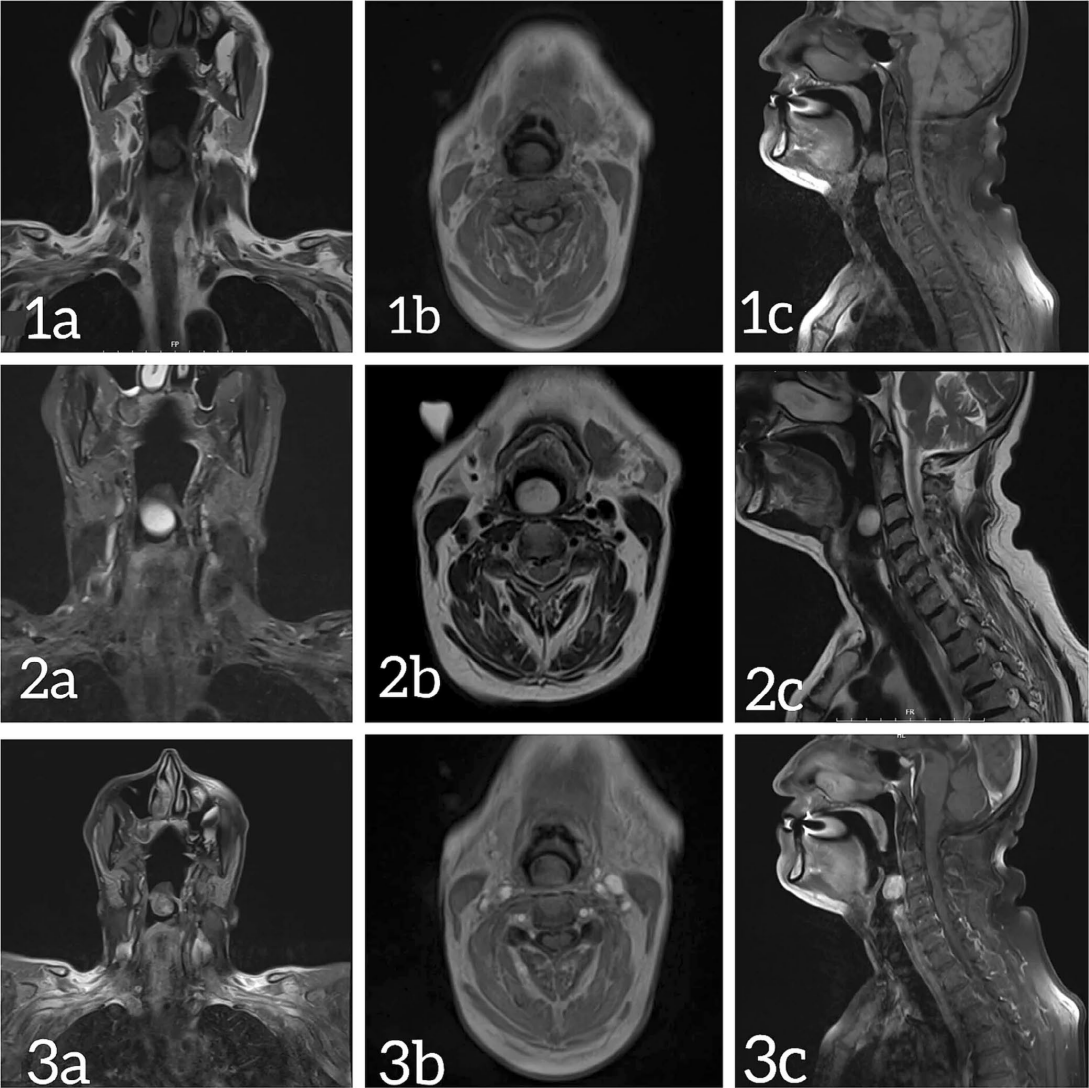
MRI of the neck demonstrates a well‐circumscribed, heterogeneous lesion in the posterior hypopharynx. Images 1, 2, and 3 represent the T1‐weighted, T2‐weighted, and gadolinium‐enhanced T1‐weighted sequences, respectively. Panels a, b, and c show the coronal, axial, and sagittal planes, respectively.

A neck MRI demonstrated a well‐defined, round, heterogeneous lesion from the posterior hypopharyngeal wall. The lesion exhibited hypointense signal on T1‐weighted imaging and high signal intensity on T2‐weighted imaging. Heterogeneous enhancement was observed on post‐ gadolinium contrast T1‐weighted images (Figure 2). No further masses or lesions suspicious for multifocal disease or neurocutaneous syndromes were seen.

Given the clinical and radiologic findings, a differential diagnosis including fibroma, papilloma, lipoma, benign salivary gland tumors, neurofibroma, schwannoma, lymphangioma, and, less likely, low‐grade malignancy was considered. However, schwannoma is by far the most likely diagnosis, particularly since hoarseness could be attributed to involvement of the recurrent laryngeal nerve. Direct laryngoscopy with excisional biopsy under general anesthesia was planned.

### 2.2 Surgical Management

**FIGURE 3:**
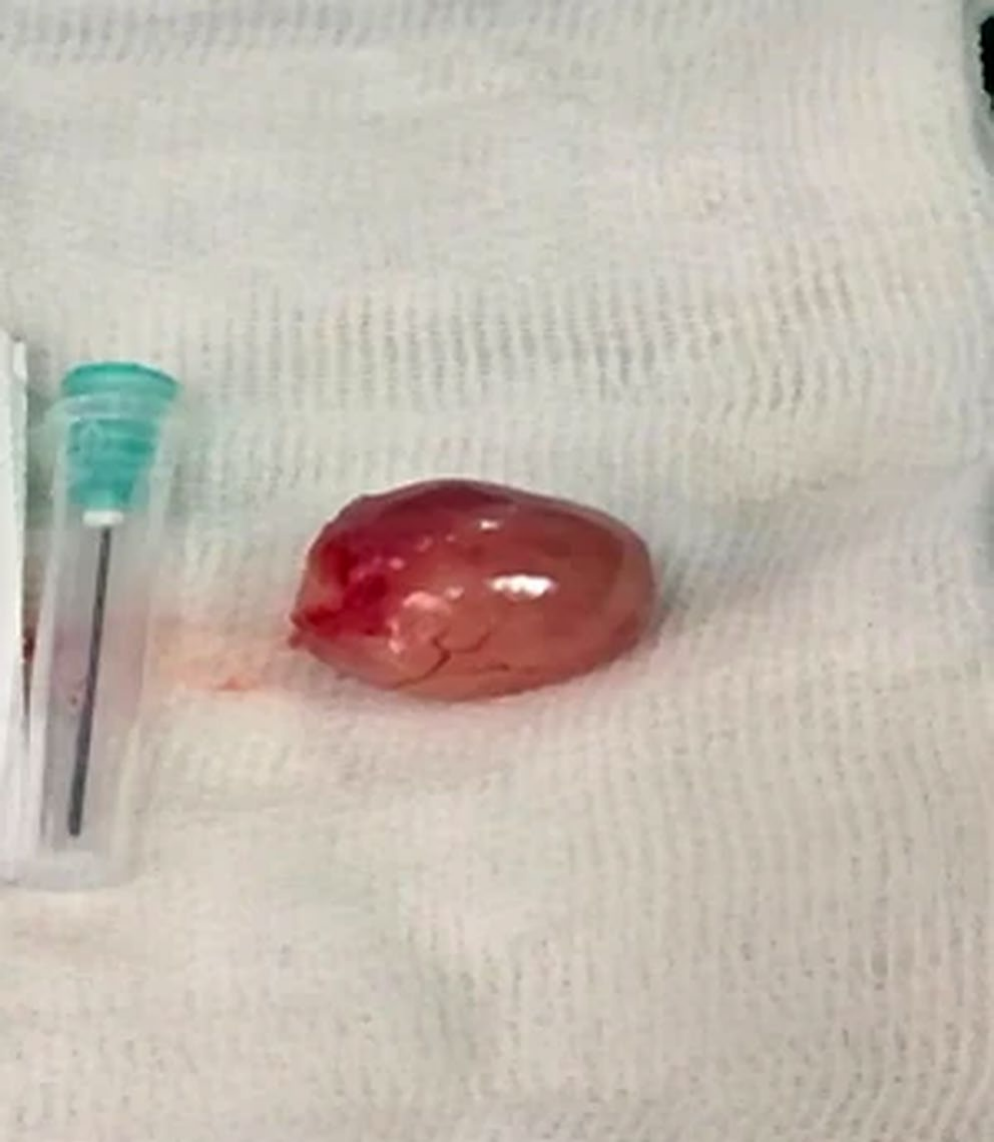
The gross specimen of the excised hypopharyngeal mass.

Transoral exposure of the hypopharynx was achieved using a supporting laryngoscope. A vertical mucosal incision over the visible mass revealed a yellow‐white, well‐encapsulated, and soft tumor within the submucosa. Blunt dissection permitted release of the mass from the underlying inferior constrictor muscle and overlying mucosa without significant bleeding. The lesion was removed en bloc (Figure 3). The resultant mucosal defect was primarily closed with absorbable sutures. The patient was extubated uneventfully and observed overnight.

### 2.3 Postoperative Course

The patient resumed oral feeding on postoperative day one and was discharged on day two. His hoarseness and dysphagia resolved completely. At 6‐month follow‐up, rigid laryngoscopy and clinical examination demonstrated no evidence of recurrence.

### 2.4 Histopathological Evaluation

**FIGURE 4:**
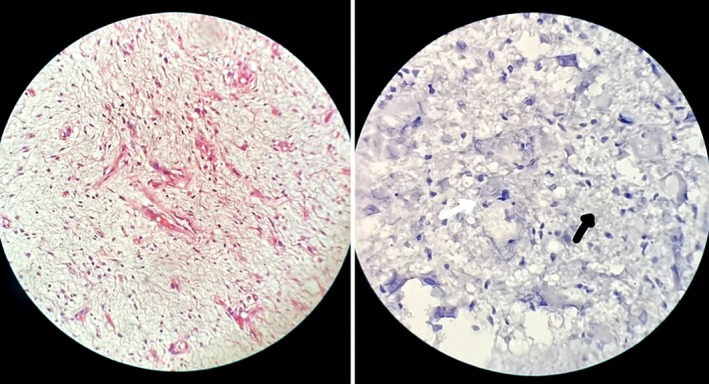
Photomicrograph (hematoxylin–eosin stain) showing interlacing spindle cells (arrow) in a myxoid stroma (black arrow), with wavy nuclei characteristic of neurofibroma.

Grossly, the specimen measured 3.1 × 1.8 × 1.7 cm, with a tan‐white, whorled cut surface. Histologically, the lesion was unencapsulated and exhibited interlacing fascicles of spindle cells embedded in a myxoid matrix with wavy nuclei, interspersed collagen bundles, and sparse mast cells (Figure 4). No nuclear atypia or mitoses were observed. Immunohistochemistry showed weak and patchy staining of tumor cells for S‐100 protein, CD34, and vimentin, while epithelial membrane antigen (EMA) and desmin were negative. In Neurofibroma, immunohistochemical evaluation demonstrates S‐100 protein expression that is typically positive but weaker and patchy, reflecting the heterogeneous cellular composition of the lesion. CD34 shows consistent positivity, highlighting the presence of fibroblastic and stromal components. Epithelial membrane antigen (EMA) may exhibit focal positivity in perineurial cells, supporting their contribution to the tumor architecture (Table 1). In contrast, schwannoma is a benign, well‐encapsulated tumor composed entirely of Schwann cells, exhibiting characteristic Antoni A and Antoni B patterns. Unlike neurofibroma, schwannoma displaces the associated nerve rather than infiltrating it and shows strong, diffuse S‐100 positivity with SOX10 expression, while EMA is typically negative and CD34 is absent or only weakly expressed. Additionally, a hybrid nerve sheath tumor was excluded in this case due to the absence of combined histopathological and immunohistochemical features [3, 4].

**TABLE 1:** Summary of Clinical, Radiologic, and Histopathologic Features.

Feature	Finding
Age/Sex	67/Male
Symptoms	Hoarseness, dysphagia
Physical findings	No neck mass, no NF1 criteria
Endoscopic appearance	Smooth submucosal mass, posterior hypopharynx
Imaging	Heterogeneous, well‐circumscribed, T1 (low), T2 (high)
Surgical approach	Transoral excision
Intraoperative findings	Easily dissected
Histology	Unencapsulated, Spindle cells, myxoid matrix, (patchy) S‐100+, CD34+
Postop outcome	Resolved, no recurrence at 6 months

## 3 Discussion

Solitary neurofibromas of the hypopharynx are distinctly rare entities. Neurofibromas represent a spectrum of benign nerve sheath tumors, with three clinicopathologic subtypes: localized (solitary), plexiform, and diffuse. While about one‐quarter of all neurofibromas are located in the head and neck region, solitary lesions in this area are uncommon. Hypopharyngeal localization is extraordinary. In our literature review, three similar cases have been identified and presented in the final section of the Discussion [5, 6, 7, 8].

### 3.1 Clinical Significance and Differential Diagnosis

Neurofibromatosis type 1 (NF1, classical/germline form) is currently diagnosed based on the updated NIH 2021 criteria, according to which the diagnosis is established when at least two of the following features are present: six or more café‐au‐lait macules, axillary or inguinal freckling, two or more cutaneous or subcutaneous neurofibromas or one plexiform neurofibroma, optic pathway glioma, two or more Lisch nodules or choroidal abnormalities, characteristic osseous lesions such as sphenoid dysplasia or tibial pseudoarthrosis, a confirmed pathogenic NF1 gene variant, or a first‐degree relative with NF1 [9]. In the clinical evaluation of the patient, there was no evidence supporting classical Neurofibromatosis type 1 (NF1) or its mosaic form. The patient lacked multiple systemic or cutaneous manifestations characteristic of NF1, including the absence of multifocal lesions and other diagnostic criteria, and the distribution pattern was neither diffuse nor segmental. Furthermore, the observed findings were not consistent with mosaic NF1, which typically presents with localized or unilateral involvement following a segmental distribution. Accordingly, NF1 was excluded, and the findings were most consistent with a solitary neurofibroma, considered a benign, isolated lesion unrelated to systemic neurofibromatosis syndromes.

The non‐specificity of clinical manifestations in hypopharyngeal neurofibroma is notable. Symptoms such as progressive hoarseness, dysphagia, foreign body sensation, or even subtle aspiration are invariably due to local mass effect from tumor growth, with systemic signs typically absent. Diagnosis is often delayed as early symptoms mimic chronic pharyngitis or reflux. Examination may easily overlook submucosal pathology unless specifically sought.

Notably, schwannomas are typically encapsulated and often remain attached to a parent nerve, while neurofibromas are usually non‐encapsulated and blend imperceptibly with nerve fibers. In the present case, the lesion was non‐encapsulated and no distinct parent nerve could be identified intraoperatively or histologically to allow separation of the mass. This lack of encapsulation and the apparent blending with surrounding nerve fibers favor a diagnosis of neurofibroma rather than schwannoma. Preoperative biopsy is not routinely performed due to the risk of hemorrhage and poor diagnostic yield [10, 11].

### 3.2 Pathological Features and Diagnosis

Definitive diagnosis rests upon histopathologic and immunophenotypic evaluation. Neurofibromas are characterized by a variable admixture of Schwann cells (S‐100 positive), fibroblasts, and perineurial cells within a loose myxoid background. Immunohistochemistry is crucial: neurofibromas are patchy S‐100 and CD34 positive, but typically EMA and desmin negative, distinguishing them from meningioma, myogenic tumors, and schwannoma. Schwannomas show uniform, intense S‐100 staining and are more likely to be encapsulated. The absence of nuclear atypia, hypercellularity, and mitoses helps exclude malignant peripheral nerve sheath tumors. Given these distinguishing features, direct excision and full pathological examination remain the gold standard for diagnosis [2, 3, 4, 5, 6, 7, 8, 9, 10, 11, 12].

### 3.3 Management: Surgical Approach and Outcomes

Complete surgical excision is the preferred and definitive treatment. The choice of approach is determined by the tumor's size, location, and relationship to the critical structures surrounding it. Historically, open external approaches, sometimes with tracheostomy, were advocated for hypopharyngeal tumors. However, with advances in endoscopic instrumentation and improved intraoperative visualization, transoral approaches (direct laryngoscopy, transoral robotic surgery) have become feasible and preferred for well‐circumscribed, accessible lesions. This method offers excellent exposure, avoids external incisions, preserves swallowing and voice, and minimizes morbidity. Recurrence following complete resection is exceedingly rare. In this case, the unencapsulated nature and lack of infiltration facilitated clean removal with primary mucosal closure, permitting a rapid and complete functional recovery [13].

### 3.4 Differential Diagnosis: Schwannoma, Minor Salivary Gland Tumor

A key diagnostic challenge is distinguishing neurofibroma from schwannoma, as both are S‐100‐positive nerve sheath tumors. Schwannoma is more likely to be encapsulated, to display “Antoni A and B” histologic areas, and usually arises from a single fascicle. Neurofibromas are composed of a more heterogeneous mixture of cells and are unencapsulated. Rarer still, low‐grade salivary neoplasms, lymphangiomas, or even granular cell tumors can present similarly, mandating thorough histopathological work‐up [2, 3, 4, 5, 6, 7, 8, 9, 10, 11, 12, 13, 14, 15].

### 3.5 Relevance to Existing Literature and Reporting Standards

Based on our search of PubMed and Google Scholar, published reports—including recent case series and the available literature—indicate that hypopharyngeal neurofibromas are rare and most commonly occur in adults between 40 and 70 years of age, without sex predilection. Clinical management is best guided by tumor size, symptoms, and risk to adjacent structures. The literature underscores the importance of individualized treatment planning; both complete excision and careful surveillance for recurrence are paramount [16]. A case reported by Li et al. [1] bears the closest macroscopic and microscopic resemblance to our present case. Their report described a postcricoid mass that was confirmed by postoperative pathological examination to be a solitary neurofibroma [1]. A relevant 2022 case involved an elderly male with a supraglottic postcricoid mass, which was postoperatively diagnosed as a solitary fibrous tumor. Notably, the cited article reported a 6% incidence of similar tumors in the head and neck region, a statistically noteworthy figure [17, 18]. Similarly, a 2013 study first reported a postcricoid region mass. Our analysis confirms that such tumors remain very rarely reported in the literature [15, 16, 17, 18, 19, 20] (Table 2).

**TABLE 2:** Summary of reported cases of hypopharyngeal neurofibroma in the literature.

Author	Sex	Age	Tumor location	Surgical approach	Outcome
Our study	Male	67	Postcricoid region	Transoral excision	No recurrence reported during follow‐up
Kiakou et al. 2022	Female	47	Postcricoid region	Transoral excision	No recurrence reported during follow‐up
Li et al. 2021	Male	58	Postcricoid region	Transoral excision	No recurrence reported during follow‐up
Cervenka et al. 2013	Female	62	Postcricoid region	Transoral excision	No recurrence reported during follow‐up
Hanna et al. 2011	Male	54	Right pyriform sinus	Transoral excision	No recurrence reported during follow‐up
Mussak et al. 2005	Male	67	Lateral hypopharyngeal	Transcervical excision	No recurrence reported during follow‐up

## 4 Conclusion

Solitary neurofibroma of the hypopharynx is an exceptional finding that should be considered in the differential diagnosis of slowly progressive upper aerodigestive symptoms with submucosal masses. Careful clinical, endoscopic, and radiologic evaluation allows precise preoperative characterization. Transoral surgical excision offers a function‐sparing and definitive treatment, and immunohistochemical analysis confirms the diagnosis. Awareness of this rare entity may facilitate early recognition and optimal management, minimizing morbidity and avoiding unnecessary radical surgery.

## Author Contributions

**Vahid Ghasem Amooeian:** data curation, project administration, writing – original draft. **Matin Ghazizadeh:** conceptualization, writing – review and editing.

## Funding

The authors have nothing to report.

## Ethics Statement

The present study complies with ethical and research standards involving humans. This article does not contain any studies involving animals performed by any of the authors.

## Consent

Written informed consent was obtained from the patient for publication of this case report and the accompanying images A copy of the written consent is available for review by the editor‐in‐chief of this journal.

## Conflicts of Interest

The authors declare no conflicts of interest.

## Data Availability

Data in the current study are available from the corresponding author on reasonable request.
